# Association between blood groups and COVID-19 outcome in Iranian patients

**DOI:** 10.2217/fvl-2021-0090

**Published:** 2021-09-22

**Authors:** Ali Asghar Ayatollahi, Bahman Aghcheli, Abolfazl Amini, Hasan Nikbakht, Parastoo Ghassemzadehpirsala, Emad Behboudi, Abdolhalim Rajabi, Alireza Tahamtan

**Affiliations:** 1 ^1^Laboratory Sciences Research Center, Golestan University of Medical Sciences, Gorgan, 4934174513, Iran; 2 ^2^Department of Microbiology, Faculty of Medicine, Golestan University of Medical Sciences, Gorgan, 4934174515, Iran; 3 ^3^Department of Medical Biotechnology, Faculty of Advanced Technologies in Medicine, Golestan University of Medical Sciences, Gorgan, 4934174516, Iran; 4 ^4^Faculty of Medical, Golestan University of Medical Sciences, Gorgan, 4934174515, Iran; 5 ^5^Biostatistics & Epidemiology Department, Faculty of Health, Environmental Health Research Center, Golestan University of Medical Sciences, Gorgan, 4934174518, Iran; 6 ^6^Infectious Diseases Research Center, Golestan University of Medical Sciences, Gorgan, 4934174510, Iran

**Keywords:** ABO, blood groups, COVID-19, Iran, Rh, SARS-CoV-2

## Abstract

**Aim:** Many factors have been speculated to explain the COVID-19 complex clinical phenotype. Due to the inconsistent data published on blood groups and COVID-19, we conducted a study on Iranian patients to further assess this association. **Materials & methods:** This retrospective study was conducted on data collected from confirmed COVID-19 hospitalized patients during March and December 2020 in a referral hospital for COVID-19, 5 Azar Hospital, Gorgan, north of Iran. A total of 1554 confirmed COVID-19 cases were enrolled in the study with blood group (ABO and Rh), demographic, and clinical data available. **Results:** Of 1554 patients, 1267 and 287 cases had recovered and deceased (due to COVID-19) outcomes, respectively. Most of the cases had O+ (29.6%), the least number had AB- (0.5%), and most of the deceased cases had O+ blood types (31.4%). Logistic regression analysis revealed that groups A- and B- had higher and groups B+, AB+, O+ and O- had lower odds of death than the A+ group. **Conclusion:** This study indicates that blood types may be related to the clinical outcome of COVID-19. Further studies with a large cohort for multiple people are required to validate this association.

COVID-19, an infectious disease caused by SARS-CoV-2, emerged in late November 2019 in Wuhan, China, and has become a pandemic disease. The WHO has identified the disease as a public health priority of international concern [1]. As an emerging virus, all people may be susceptible to SARS-CoV-2 infection, but the clinical course and outcome of COVID-19 vary significantly among individuals. While most infected cases present asymptomatic or mild symptoms, a small number are severely affected by COVID-19 [2]. It is unclear why severe disease and death occur only in a small subset of COVID-19 patients [3]. Despite efforts to elucidate mechanisms for differential clinical responses to SARS-CoV-2 infection, they remain largely elusive.

Although older patients with predisposing risk factors are more susceptible to severe forms of COVID-19, several cases of severe infection occur among previously healthy individuals [4]. Many factors (related to host, virus and environment) have been speculated to explain the COVID-19 complex clinical phenotype, but the exact contributions of each factor remain contentious [3]. It is well accepted that host differences (mainly genetic makeup) could dictate any viral infection’s clinical course and outcome [3,5]. People are different in many aspects, and we are in the preliminary stage to characterize stimulating factors for this complex disease. Since we have to live with SARS-CoV-2 for a long time, finding specific factors associated with the complicated disease outcome is very important.

The association between blood groups and susceptibility to some viral infections has been well established [6–11]. The blood group antigens are cell-surface glycoproteins present principally on erythrocytes that may affect infectious diseases [12]. During the current pandemic, several studies have investigated the association of blood groups and COVID-19 [13–18]. Some studies suggested that patients with blood group A are more susceptible to severe infection, but there are other controversial results [19–24]. More studies are needed to confirm the association between blood grouping and COVID-19 susceptibility and severity. Here, we investigated the association between blood groups (ABO and Rh) and COVID-19 outcomes in Iranian hospitalized patients.

## Materials & methods

This retrospective study was conducted on data collected from patients hospitalized during March and December 2020 in a referral hospital for COVID-19, 5 Azar Hospital, Gorgan, north of Iran. A total of 1554 confirmed COVID-19 cases were enrolled in the study with blood group (ABO and Rh), demographic, and clinical data available. All COVID-19 patients had been confirmed by reverse transcriptase real-time PCR assay targeting the SARS-CoV-2 nucleoprotein (N) and ORF1ab genes (Pishtazteb, Iran). Data such as blood group, gender, age, hospitalized ward and disease outcome was collected from the patients’ case report forms. Because the frequency of participants in different age groups was different, the patients were classified into different age groups according to the literature. The patients were also classified into different hospitalized wards. Two outcomes, recovered and deceased (due to COVID-19), were examined in this study. All of the patients were from Golestan Province and had the same geographical origin.

Analyses were performed using SPSS version 25.0 (IBM Corp., NY, USA). Data were presented in proportions. We performed a chi-square test to compare the proportion of blood groups, age group, sex and hospitalization wards between the recovered and deceased groups. Multiple logistic regression was computed to assess the odds of death (dependent variable). Independent variables included the blood groups, age group, sex and hospitalization wards. The reported p-value is two-sided, and p-values less than 0.05 were considered statistically significant.

## Results

Of 1554 patients, 1267 and 287 cases had recovered and deceased outcomes, respectively. Of all cases, 814 (52.4%) and 740 (47.6%) were male and female, respectively. The mean age of all patients was 56.66 ± 17.15 (median: 57 years; range: 1–101 years). The patients were distributed into five groups: 1–29 years, 30–39 years, 40–49 years, 50–59 years and ≥60 years. Hospital wards were ICU (intensive care unit)/CCU (cardiac care unit)/emergency, infectious disease, post-ICU/isolated room and other wards. Details of the distribution of the demographic, gender, age, hospitalization ward and blood groups of all, recovered and deceased cases are presented in Table 1.

**Table 1.** Demographic, sex, age, blood group and hospitalization wards of all, recovered and deceased cases.

Variables	All cases (%)	Recovered (%)	Deceased (%)	p-value
Sex				
Female	740 (47.6)	606 (47.8)	134 (46.7)	
Male	814 (52.4)	661 (52.2)	153 (53.3)	
Total	1554 (100)	1267 (81.5)	287 (18.5)	0.354

**Age (years)**				
1–29	92 (5.92)	85 (6.71)	7 (2.44)	
30–39	174 (11.20)	159 (12.55)	15 (5.23)	
40–49	279 (17.95)	254 (20.05)	25 (8.71)	
50–59	314 (20.21)	271 (21.39)	43 (14.98)	
≥60	695 (44.72)	498 (39.31)	197 (68.64)	<0.001

**Blood group**				
A+	427 (27.5)	342 (27)	85 (29.6)	
A-	45 (2.9)	31 (2.4)	14 (4.9)	
B+	401 (25.8)	353 (27.9)	48 (16.7)	
B-	35 (2.3)	18 (1.4)	17 (5.9)	
AB+	105 (6.8)	86 (6.8)	19 (6.6)	
AB-	7 (0.5)	7 (0.6)	0 (0.0)	
O+	460 (29.6)	370 (29.2)	90 (31.4)	
O-	74 (4.8)	60 (4.7)	14 (4.9)	<0.001

**Hospitalization wards**				
Infectious Disease	1102 (70.91)	1078 (85.08)	24 (8.36)	
ICU/CCU/Emergency	288 (18.53)	52 (4.10)	236 (82.23)	
Post-ICU/isolated room	74 (4.76)	54 (4.26)	20 (6.97)	
Other wards	90 (5.79)	83 (6.55)	7 (2.44)	<0.001

CCU: Cardiac care unit; ICU: Intensive care unit.

In this study, there was no significant association between gender and the outcome of COVID-19. The data showed a significant association between age and the outcome of the disease. The number of COVID-19 cases and deaths gradually increased with age, so the odds of the deaths were higher in the age group over 60 years compared with the reference group. We found a significant association between hospitalization ward and outcome of the disease. The odds of death were higher in those hospitalized in the ICU/CCU/emergency wards than in the infectious disease group. Most of the COVID-19 cases in this study had O+, the least had AB- and most deaths had O+ blood types. Logistic regression analysis revealed that odds of death in group A- versus A+ were 1.81-times higher, though it was not significant. Blood type B+ had lower odds of death than A+. Patients with type B-, 3.8-times more likely for COVID-19 death than the A+ blood group. Patients with the AB+ group had lower odds of death than the A+. Finally, group O+ and O- had a lower odds of death than the A+ blood group. The details association of sex, age, blood group and hospitalization wards of COVID-19 patients with risk of death are presented in Table 2.

**Table 2.** Association of sex, age, blood group and hospitalization wards of COVID-19 patients with risk of death.

Variables	All cases (%)	OR (CI: 95%)	p-value
Sex			
Female	740 (47.6)	Ref.	0.27
Male	814 (52.4)	1.15 (0.88–1.51)	
Total	1554 (100)		

**Age (years)**			
1–29	92 (5.92)	Ref.	
30–39	174 (11.20)	1.16 (0.45–2.96)	0.75
40–49	279 (17.95)	1.22 (0.51–2.93)	0.65
50–59	314 (20.21)	1.97 (0.85–4.55)	0.11
≥60	695 (44.72)	4.95 (2.24–10.91)	<0.001

**Blood group**			
A+	427 (27.5)	Ref.	
A-	45 (2.9)	1.817 (0.925–3.566)	0.083
B+	401 (25.8)	0.547 (0.372–0.803)	0.002
B-	35 (2.3)	3.8 (1.879–7.683)	<0.001
AB+	105 (6.8)	0.888 (0.512–1.541)	0.675
AB-	7 (0.5)	.	
O+	460 (29.6)	0.978 (0.702–1.362)	0.899
O-	74 (4.8)	0.938 (0.500–1.759)	0.844

**Hospitalization wards**			
Infectious disease	1102 (70.91)	Ref.	
ICU/CCU/emergency	288 (18.53)	203.85 (123.178–337.362)	<0.001
Post-ICU/isolated room	74 (4.76)	16.635 (8.655–31.972)	<0.001
Other wards	90 (5.79)	3.788 (1.585–9.051)	0.003

CCU: Cardiac care unit; ICU: Intensive care unit.

## Discussion

This study indicates that blood types may be related to the clinical outcome of COVID-19. The main findings of this study are: no significant association between gender and outcome of COVID-19, a significant association between age and the outcome of the disease, a significant association between hospitalization ward and outcome of COVID-19 and blood groups A- and B- have higher and groups B+, AB+, O+ and O- have lower odds of death than the A+ group (Table 1 & 2).

Previous studies demonstrated that male cases have an increased risk of death compared with females [25]. However, in this study, there was no significant association between gender and the outcome of COVID-19. Studies also demonstrated that age could be considered as a death-associated risk factor for COVID-19 [3,26]. Most of the data in this context agree that the infection and death were mostly observed in cases with advanced ages. Our data showed a significant association between age and the outcome of the disease. The number of COVID-19 cases and deaths gradually increased with age, so the odds of the deaths were higher in the age group over 60 years. This finding might be expected because elderlies tend to have a higher prevalence of comorbidities such as diabetes, heart disease, hypertension, chronic respiratory diseases and they have a reduced immune response. A significant association between hospitalization ward and outcome of the disease was found, and also odds of death were higher in those hospitalized in the ICU/CCU/emergency wards. This finding also might be expected because critically ill COVID-19 patients are hospitalized in these wards.

Regarding blood groups, our data showed that odds of death in the group A- versus A+ were 1.81-times higher, and patients with type B-, 3.8-times more likely for COVID-19 death than the A+ blood group. However, other blood groups such as B+, AB+, O+ and O- had a lower odds of death than the A+ blood group. It is not yet clear which blood type is most likely to be susceptible to COVID-19. Some studies considered that people with blood type A to be the most susceptible to the disease, and people with blood type O are less likely to develop the disease [13–15,17–20,24,27–55]. But other studies reported conflicting results [22,23,56,57], and some did not find a significant relationship between blood type and COVID-19 [16,21,58–60]. Moreover, studies reported that individuals with Rh- blood type had a lower risk of infection, intubation and death, and cases with Rh+ were more sensitive to COVID-19 [24,27–29,56,57]. However, a study from Iran did not find any relation between COVID-19 and Rh type [30]. Another study in Turkey also did not report a relationship between Rh blood groups and any impact on the rate of hospital admission, ICU stay, mechanical ventilation support and case fatality rate [31]. The literature studies regarding the association between blood types and COVID-19 are summarized in Table 3.

**Table 3.** The literature regarding the association between blood types and COVID-19.

Study (year)	Countries	Sample size	Main findings	Ref.
Zhang *et al.* (2021)	Literature review	23 study	Greatest risk for susceptibility to COVID-19 infection was found among individuals with blood type A	[13]
Kabrah *et al.* (2021)	Systematic review and meta-analysis	16 study	Blood group A cases were vulnerable to COVID-19 infection, and blood type AB linked to a lower risk of COVID-19 infection	[14]
Liu *et al.* (2020)	Systematic review and meta-analysis	10 study	Individuals with blood groups A and B were more susceptible to COVID-19 infection, whereas the blood group O appeared to be protective	[15]
Franchini *et al.* (2021)	Systematic literature review and meta-analysis	21 study	Blood group O individuals were less susceptible to SARS-CoV-2 infection compared with those in the non-O group. No evidence was found indicating an effect of the O type on disease severity in SARS-CoV-2 infection	[17]
Mahmud *et al.* (2021)	Bangladesh	438	Although ABO blood groups were not associated with the presentation or recovery period of COVID-19, patients with blood group A had delayed seroconversion	[18]
Garg *et al.* (2021)	India	383	COVID-19 patients with A and B blood groups exhibit greater disease severity. There are no differences between distribution of Rh (D) +ve type and Rh (D) -ve in severe, moderate and mild patients	[19]
Kim *et al.* (2021)	Review	9 study	Blood type A patients had higher risk and blood type O patients had lower risk of becoming infected with SARS-CoV-2	[20]
Samra *et al.* (2021)	Saudi Arabia	507	The incidence, severity, and mortality of COVID-19 were common in nonblood group O, while blood group O was protected against COVID-19	[24]
Zietz *et al.* (2020)	United States	14112	Blood type O was protective against SARS-CoV-2 infection compared with non-O blood. Blood type A patients had lower risk of intubation and death compared with blood type O patients, whereas blood type AB had higher risk of both intubation and death. The Rh- patients had a 2.7% lower risk of initial infection after adjustment, and also lower risk for both intubation and death	[27]
Ray *et al.* (2020)	Canada	225,556	Blood type A and AB had higher risk of severe illness or death. B+ was associated with higher odds of testing positive, whereas O- was associated with lower infection rate. The Rh+ cases had higher risk of severe illness or death	[28]
Taha *et al.* (2020)	Sudan	557	The O+ blood group had the lowest risk of having severe symptoms, and A+ individuals were the most vulnerable when exposed to the virus	[29]
Abdollahi *et al.* (2020)	Iran	397	Blood group A was significantly associated with a higher risk of infection and blood group O was associated with a lower risk. The Rh blood group phenotype was not statistically significant in determining a patient’s vulnerability	[30]
Dal *et al.* (2021)	Turkey	39,850	ABO and Rh blood groups did not have any impact on the rate of hospital admission, hospital and ICU stay, mechanical ventilation support and case fatality rate. Blood group A was found to be related with increased rate of ICU admission	[31]
Fan *et al.* (2020)	China	Cases: 105 Controls: 103	Blood type A patients had higher risk and blood type O patients had lower risk of becoming infected with SARS-CoV-2	[32]
Zhao *et al.* (2020)	China	2173	Blood type A patients had higher risk and blood type O patients had lower risk of becoming infected with SARS-CoV-2	[33]
Golinell *et al.*	Meta-analysis	Cases: 7503 Controls: 2,962,160	Blood type A patients had higher risk and blood type O patients had lower risk of becoming infected with SARS-CoV-2	[34]
Barnkob *et al.* (2020)	Denmark	Cases: 7422 Controls: 466,232	Blood type O was protective against SARS-CoV-2 infection compared with non-O blood type	[35]
Wu *et al.* (2020)	Systematic review and meta-analysis	31,100	Blood type A patients had higher risk and blood type O patients had lower risk of becoming infected with SARS-CoV-2	[36]
Padhi *et al.* (2020)	India	277,649	Blood type O was protective against COVID-19 death, while blood type B was more strongly correlated with death	[37]
Li *et al.* (2020)	China	Cases: 2153 Controls: 3694	Blood type A patients had higher risk and blood type O patients had lower risk of becoming infected with SARS-CoV-2	[38]
Goker *et al.* (2020)	Turkey	186	Blood group A might have a role in increased susceptibility to the COVID-19 infection, and the blood group O might be somewhat protective	[39]
Leaf *et al.* (2020)	USA	3239	Type A blood may be a risk factor for COVID-19-related critical illness among White patients, and that type O blood may be protective	[40]
Zeng *et al.* (2020)	China	97	Blood type A individuals were more sensitive to SARS-CoV-2 infection. Blood type distribution was not a relevant factor of ARDS, AKI and mortality in COVID-19 patients	[41]
Chegni *et al.*	Iran	76	Blood group A was significantly associated with a higher risk of infection and blood group O was associated with a lower risk of infection	[42]
Zhang *et al.* (2020)	China	134	Blood group A was significantly associated with a higher risk of infection and blood group O was associated with a lower risk of infection	[43]
Roberts *et al.* (2020)	USA	2417	Blood group A was significantly associated with a higher risk of infection	[44]
Marcos *et al.* (2020)	Spain	226	Blood group A was significantly associated with a higher risk of infection and blood group O was associated with a lower	[45]
Franchini *et al.* (2020)	Italy	447	Blood type O may be protective against COVID-19	[46]
Aljanobi *et al.* (2020)	Saudi-Arabia	72	Blood group A was significantly associated with a higher risk of infection and blood group O was associated with a lower	[47]
Valenti *et al.* (2020)	Italy/Spain	505	Blood group A was associated with higher risk of severe COVID-19 than group O, while group B was not. Increased risk of severe disease was observed in carriers of the AB group	[48]
Hoiland *et al.* (2020)	Canada	95	A greater proportion of blood group A or AB patients required mechanical ventilation and CRRT compared with blood group O or B patients	[49]
Wu *et al.* (2020)	China	187	Blood type A patients had higher risk and blood type O patients had lower risk of becoming infected with SARS-CoV-2. There is no correlation between blood type and COVID-19 severity or mortality	[50]
Diaz *et al.* (2021)	Spain	854	Blood group A was significantly associated with a higher risk of infection and blood group O was associated with a lower	[51]
Ahmed *et al. *(2021)	UK	86	Blood group A women had a significantly higher relative risk of developing COVID-19 infection	[52]
Solmaz *et al. *(2021)	Turkey	1667	Blood group A was significantly associated with a higher risk of infection and blood group O was associated with a lower	[53]
Cordero *et al. *(2021)	International	9859	Blood group A was significantly associated with a higher risk of infection and blood group O was associated with a lower	[54]
Solhpour *et al. *(2020)	Iran	93	Blood group type A is usually more susceptible to be involved by severe form of the COVID-19	[55]
Almadhi *et al. *(2021)	Bahrain	Cases: 2334 Controls: 4985	Blood group AB was associated with a decreased risk of infection, while blood group B was associated with an increased risk. No association between blood group A and risk of COVID-19 infection was found	[22]
Ishaq *et al.* (2021)	Pakistan	1067	Hospital stay, severity of disease and mortality were associated with blood group A	[23]
Niles *et al.* (2021)	USA	34,178	The SARS-CoV-2 positivity rate was significantly higher among type O and Rh+ patients	[56]
Latz *et al.* (2020)	USA	1289	Blood type B and AB were associated with higher risk of testing positive for COVID-19. Blood type O was associated with lower risk of testing positive. There is no correlation between blood type and COVID-19 intubation and death. Individuals with Rh- blood type were less susceptible to infection by SARS-CoV-2	[57]
Bai *et al.*	Systematic review	24 study	There is no true relationship between ABO blood type and COVID-19 infection, severity or mortality	[16]
Anderson *et al. *(2021)	USA	Cases: 11,468 Controls: 96,328	No ABO associations were found with either disease susceptibility or severity	[21]
Levi *et al.* (2021)	Brazil	2037	Absence of a relationship between ABO blood type and susceptibility to SARS-CoV-2 infection	[58]
Boudin *et al.* (2020)	France	1769	Absence of a relationship between ABO blood type and susceptibility to SARS-CoV-2 infection	[59]
Dzik *et al.* (2020)	USA	957	No association was found between ABO type and death among individuals hospitalized with COVID-19	[60]
Samra *et al.* (2021)	Saudi Arabia	507	The severity of COVID-19 infection was common in Rh+ patients and Rh- patients	[24]
Garg *et al.* (2021)	India	383	There was no differences between distribution of Rh (D) +ve type and Rh (D) -ve in severe, moderate and mild patients	[19]

AKI: Acute kidney injury; ARDS: Acute respiratory distress syndrome; CRRT: Continuous renal replacement therapy; ICU: Intensive care unit.

The discrepancy between studies may be due to the different sample sizes, heterogeneity of ABO between populations or geographical areas, differences in genetic background and differences in viral strains. Variation between blood group phenotypes in countries and different genetics may affect heterogeneity of COVID-19 clinical phenotypes [21,61]. Susceptibility to SARS-CoV-2 infection and ABO blood system might be for some reasons: blood group O patients had natural anti-A and anti-B antibodies that could be partially protective against SARS-CoV-2 virions, carbohydrate–carbohydrate interactions, which could maximize or minimize the virus spike protein binding to the host cell, furin levels might be reduced in blood type O individuals, so the infectivity of virus is reduced, the levels of C-reactive protein and alkaline phosphatase appear to be higher in blood group A individuals in comparison with blood group O and microbiota trigger the synthesis of anti-A and anti-B antibodies [61,62].

## Conclusion

Finding high-risk groups for severe infection and death are important to manage the current pandemic disease. The results of such studies could lead to individuals with a higher risk of severe infection being vaccinated earlier or monitored more closely and treated earlier. Together, these findings suggest that the blood group may interplay with susceptibility to SARS-CoV-2 infection and clinical course of COVID-19; however, the mechanism(s) is subjected to speculations. The limitation of this study was the small sample size, and further studies with a large cohort for multiple people are required to validate this association.

**Summary points**

- Due to the inconsistent data published on blood groups and COVID-19, we conducted a study on Iranian patients to further assess this association.
- A total of 1554 confirmed COVID-19 cases were enrolled in the study with blood group, demographic and clinical data available.
- Of 1554 patients, 1267 and 287 cases had recovered and deceased outcomes, respectively.
- Most of the cases had O+ (29.6%), the least number had AB- (0.5%), and most of the deceased cases had O+ blood types (31.4%).
- Logistic regression analysis revealed that groups A- and B- had higher and groups B+, AB+, O+ and O- had lower odds of death than the A+ group.
- Our findings indicate that blood types may be related to the clinical outcome of COVID-19.
- Further studies with a large cohort for multiple people are required to validate this association.

## References

[1] Teymoori-Rad M, Samadizadeh S, Tabarraei A, Moradi A, Shahbaz M, Tahamtan A. Ten challenging questions about SARS-CoV-2 and COVID-19. Expert Rev. Respir. Med. 14(9), 881–888 (2020). 32536226 10.1080/17476348.2020.1782197

[2] Tahamtan A. Genetic cackground as a missing factor in COVID-19 severity. JCBR 4(3), 1–2 (2020).

[3] Samadizadeh S, Masoudi M, Rastegar M, Salimi V, Shahbaz M, Tahamtan A. COVID-19: why does disease severity vary among individuals? Respir. Med. 180, 106356 (2021). 33713961 10.1016/j.rmed.2021.106356 PMC7934673

[4] Brodin P. Immune determinants of COVID-19 disease presentation and severity. Nat. Med. 27(1), 28–33 (2021). 33442016 10.1038/s41591-020-01202-8

[5] Tahamtan A, Samadizadeh S, Rastegar M, Nakstad B, Salimi V. Respiratory syncytial virus infection: why does disease severity vary among individuals? Expert. Rev. Respir Med. 14(4), 415–423 (2020). 31995408 10.1080/17476348.2020.1724095

[6] Cheng Y, Cheng G, Chui C ABO blood group and susceptibility to severe acute respiratory syndrome. JAMA 293(12), 1447–1451 (2005). 10.1001/jama.293.12.1450-c 15784866

[7] Hutson A, Atmar R, Graham D, Estes M. Norwalk virus infection and disease is associated with ABO histo-blood group type. J. Infect. Dis. 185(9), 1335–1337 (2002). 12001052 10.1086/339883

[8] Batool Z, Durrani SH, Tariq S Association Of ABO and Rh blood group types to hepatitis B, hepatitis C, HIV and syphilis infection, a five year’ experience in healthy blood donors in a tertiary care hospital. J. Ayub. Med. Coll. Abbottabad. 29(1), 90–92 (2017). 28712183

[9] Lebiush M, Rannon L, Kark JD The relationship between epidemic influenza (A(H1N1) and ABO blood group. J. Hyg. (Lond.) 87(1), 139–146 (1981). 10.1017/s002217240006931x PMC2134079 7252135

[10] Conton B, Gevao S, Sahr F. Do ABO and rhesus blood groups affect susceptibility to, and prognosis of ebola virus infection? Virol. J. (2017) (Epub ahead of print).

[11] Sestak K. Role of histo-blood group antigens in primate enteric calicivirus infections. World J. Virol. 3(3), 18–21 (2014). 25392814 10.5501/wjv.v3.i3.18 PMC4227010

[12] Arend P. Why blood group A individuals are at risk whereas blood group O individuals are protected from SARS-CoV-2 (COVID-19) infection: a hypothesis regarding how the virus invades the human body via ABO(H) blood group-determining carbohydrates. Immunobiology 226(3), 152027 (2020). 33706067 10.1016/j.imbio.2020.152027 PMC7609233

[13] Zhang Y, Garner R, Salehi S Association between ABO blood types and coronavirus disease 2019 (COVID-19), genetic associations, and underlying molecular mechanisms: a literature review of 23 studies. Ann. Hematol. 100(5), 1123–1132 (2021). 33686492 10.1007/s00277-021-04489-w PMC7939543

[14] Kabrah S, Kabrah A, Flemban A Systematic review and meta-analysis of the susceptibility of ABO blood group to COVID-19 infection. Transfus. Apher. Sci. 60(4), 103169 (2021). 34045120 10.1016/j.transci.2021.103169 PMC8139534

[15] Liu N, Zhang T, Ma L The impact of ABO blood group on COVID-19 infection risk and mortality: a systematic review and meta-analysis. Blood Rev. 48, 100785–100785 (2021). 33309392 10.1016/j.blre.2020.100785 PMC7834371

[16] Bai Y, Yan Z, Murray EJ. Systematic review of the association between ABO blood type and COVID-19 incidence and mortality. medRxiv. (2021) (Epub ahead of print).

[17] Franchini M, Cruciani M, Mengoli C ABO blood group and COVID-19: an updated systematic literature review and meta-analysis. Blood. Transfus. 19(4), 317–326 (2021). 10.2450/2021.0049-21 PMC8297670 34059188

[18] Mahmud R, Aftab M, Binte F Association of ABO blood groups with presentation and outcomes of confirmed SARS CoV-2 infection: a prospective study in the largest COVID-19 dedicated hospital in Bangladesh. PLoS ONE 16(4), e0249252 (2021). 33826648 10.1371/journal.pone.0249252 PMC8026078

[19] Garg I, Srivastava S, Dogra V Potential association of COVID-19 and ABO blood group: An Indian study. Microb. Pathog. 158, 105008–105008 (2021). 34087389 10.1016/j.micpath.2021.105008 PMC8168328

[20] Kim Y, Christopher A, Charles S Relationship between blood type and outcomes following COVID-19 infection. Semin. Vasc. Surg. (2021) (Epub ahead of print). 10.1053/j.semvascsurg.2021.05.005 PMC8286549 34642032

[21] Anderson J, May HT, Knight S Association of sociodemographic factors and blood group type with risk of COVID-19 in a US population. JAMA Network Open. 4(4), e217429–e217429 (2021). 33818622 10.1001/jamanetworkopen.2021.7429 PMC8022215

[22] Almadhi M, Abdulrahman A, Alawadhi A, Rabaan A, Atkin S, AlQahtani M. The effect of ABO blood group and antibody class on the risk of COVID-19 infection and severity of clinical outcomes. Sci. Rep. 11(1), 5745 (2021). 33707451 10.1038/s41598-021-84810-9 PMC7952683

[23] Ishaq U, Malik A, Malik J Association of ABO blood group with COVID-19 severity, acute phase reactants and mortality. medRxiv (2021) (Epub ahead of print). 10.1371/journal.pone.0261432 PMC8670663 34905588

[24] Samra S, Habeb M, Nafae R. ABO groups can play a role in susceptibility and severity of COVID-19. Egypt. J. Bronchol. 15(1), 9 (2021).

[25] Jin J-M, Bai P, He W Gender differences in patients with COVID-19: focus on severity and mortality. Front. Public Health 8(152), (2020). 10.3389/fpubh.2020.00152 PMC7201103 32411652

[26] Collaborative G.M.R. Age and frailty are independently associated with increased COVID-19 mortality and increased care needs in survivors: results of an international multi-centre study. Age Ageing 50(3), 617–630 (2021). 33543243 10.1093/ageing/afab026 PMC7929433

[27] Zietz M, Zucker J, Tatonetti NP. Testing the association between blood type and COVID-19 infection, intubation, and death. medRxiv (2020) (Epub ahead of print). 10.1038/s41467-020-19623-x PMC7666188 33188185

[28] Ray J, Schull M, Vermeulen M, Park A. Association between ABO and Rh blood groups and SARS-CoV-2 infection or severe covid-19 illness: a population-based cohort study. Ann. Intern. Med. 174(3), 308–315 (2021). 33226859 10.7326/M20-4511 PMC7711653

[29] Taha SAH, Osman MEM, Abdoelkarim EAA. Individuals with a Rh-positive but not Rh-negative blood group are more vulnerable to SARS-CoV-2 infection: demographics and trend study on COVID-19 cases in Sudan. NMNI 38, 100763 (2020). 32983543 10.1016/j.nmni.2020.100763 PMC7505818

[30] Abdollahi A, Mahmoudi M, Mehrtash V, Jafarzadeh B, Salehi M. The novel coronavirus SARS-CoV-2 vulnerability association with ABO/Rh blood types. IJP 15(3), 156–160 (2020). 32754209 10.30699/ijp.2020.125135.2367 PMC7354076

[31] Dal M, Ata N, Altuntaş F COVID-19 clinical course and blood groups: Turkish population-based study. Turk. J. Med. Sci. (2021) (Epub ahead of print). 10.3906/sag-2101-321 33957720

[32] Fan Q, Zhang W, Li B, Li D-J, Zhang J, Zhao F. Association between ABO blood group system and COVID-19 susceptibility in Wuhan. Front. Cell. Infect. Microbiol. 10, 404 (2020). 32793517 10.3389/fcimb.2020.00404 PMC7385064

[33] Zhao J, Yang Y, Huang H Relationship between the ABO blood group and the coronavirus disease 2019 (COVID-19) susceptibility. Clin. Infect. Dis. 73(2), 328–331 (2020). 10.1093/cid/ciaa1150 PMC7454371 32750119

[34] Golinelli D, Boetto E, Maietti E, Fantini M. The association between ABO blood group and SARS-CoV-2 infection: a meta-analysis. PLoS ONE 15(9), e0239508 (2020). 32946531 10.1371/journal.pone.0239508 PMC7500631

[35] Barnkob M, Pottegård A, Støvring HE Reduced prevalence of SARS-CoV-2 infection in ABO blood group O. Blood. Adv. 4(20), 4990–4993 (2020). 33057631 10.1182/bloodadvances.2020002657 PMC7594382

[36] Wu B-B, Gu D-Z, Yu J-N Association between ABO blood groups and COVID-19 infection, severity and demise: a systematic review and meta-analysis. Infect. Genet. Evol. 84, 104485 (2020). 32739464 10.1016/j.meegid.2020.104485 PMC7391292

[37] Padhi S, Suvankar S, Dash D ABO blood group system is associated with COVID-19 mortality: an epidemiological investigation in the Indian population. Transfus. Clin. Biol. 27(4), 253–258 (2020). 32987167 10.1016/j.tracli.2020.08.009 PMC7518849

[38] Li J, Wang X, Chen J, Cai Y, Deng A, Yang M. Association between ABO blood groups and risk of SARS-CoV-2 pneumonia. Br. J. Haematol. 190(1), 24–27 (2020). 32379894 10.1111/bjh.16797 PMC7267665

[39] Göker H, Karakulak E, Demiroğlu H The effects of blood group types on the risk of COVID-19 infection and its clinical outcome. Turk. J. Med. Sci. 50(4), 679–683 (2020). 32496734 10.3906/sag-2005-395 PMC7379446

[40] Leaf R, Al-Samkari H, Brenner S, Gupta S, Leaf D. ABO phenotype and death in critically ill patients with COVID-19. Br. J. Haematol. 190(4), e204–e208 (2020). 32609874 10.1111/bjh.16984 PMC7361419

[41] Zeng X, Fan H, Lu D. Association between ABO blood groups and clinical outcome of coronavirus disease 2019: evidence from two cohorts. medRxiv (2020) (Epub ahead of print).

[42] Chegni H, Pakravan N, Saadati M, Ghaffari A, Shirzad H, Hassan Z. Is there a link between COVID-19 mortality with genus, age, ABO blood group type, and ACE2 gene polymorphism? Iran. J. Public Health. 49(8), 1582–1584 (2020). 33083341 10.18502/ijph.v49i8.3910 PMC7554399

[43] Zhang L, Huang B, Xia H. Retrospective analysis of clinical features in 134 coronavirus disease 2019 cases. IEM 148, e199–e199 (2020). 10.1017/S0950268820002010 PMC7487751 32878654

[44] Roberts G, Park D, Coignet M. AncestryDNA COVID-19 host genetic study identifies three novel loci. medRxiv (2020) (Epub ahead of print).

[45] Marcos S, Antelo M, Galbete A, Etayo M, Ongay E, Antonio García-Erce J. Infection and thrombosis associated with COVID-19: possible role of the ABO blood group. Medicina Clinica 155(8), 340–343 (2020). 10.1016/j.medcle.2020.06.013 PMC7519708 33015369

[46] Franchini M, Glingani C, Fante C. The protective effect of O blood type against SARS-CoV-2 infection. Vox Sang. 116(2), 249–250 (2021). 32950039 10.1111/vox.13003 PMC7537255

[47] Aljanobi G, Alhajjaj A, Alkhabbaz FA, Al-Jishi J. The relationship between ABO blood group type and the COVID-19 susceptibility in Qatif Central Hospital, Eastern Province, Saudi Arabia: a retrospective cohort study. Open J. Intern. Med. 10, 232–238 (2020).

[48] Valenti L, Villa S, Baselli G. Association of ABO blood group and secretor phenotype with severe COVID-19. Transfusion 60(12), 3067–3070 (2020). 33009662 10.1111/trf.16130 PMC7675339

[49] Hoiland R, Fergusson N, Mitra A The association of ABO blood group with indices of disease severity and multiorgan dysfunction in COVID-19. Blood Adv. 4(20), 4981–4989 (2020). 33057633 10.1182/bloodadvances.2020002623 PMC7594392

[50] Wu Y, Feng Z, Li P, Yu Q. Relationship between ABO blood group distribution and clinical characteristics in patients with COVID-19. Clin. Chim. Acta 509, 220–223 (2020). 32562665 10.1016/j.cca.2020.06.026 PMC7832938

[51] Diaz E, lopis J, Parra R. Relationship between the ABO blood group and COVID-19 susceptibility, severity and mortality in two cohorts of patients. Blood Transfus. 19(1), 54–63 (2021). 33196417 10.2450/2020.0256-20 PMC7850930

[52] Ahmed I, Quinn L, Tan BK. COVID-19 and the ABO blood group in pregnancy: a tale of two multiethnic cities. Int. J. Lab. Hematol. 43(1), e45–e47 (2021). 32996710 10.1111/ijlh.13355 PMC7537203

[53] Solmaz İ, Araç S. ABO blood groups in COVID-19 patients; cross-sectional study. Int. J. Clin. Pract. 75(4), e13927 (2021). 33296536 10.1111/ijcp.13927 PMC7883261

[54] Cordero A, Li X, Milne S. Multi-omics highlights ABO plasma protein as a causal risk factor for COVID-19. Hum. Genet. 140(6), 969–979 (2021). 33604698 10.1007/s00439-021-02264-5 PMC7892327

[55] Solhpour A, Jafari A, Pourhoseingholi M, Soltani F. Corona COVID-19 virus and severe hypoxia in young patients without underlying disease: high prevalence rate with blood group A. Trends Anaesth. Crit. Care. 34, 63–64 (2020). 10.1016/j.tacc.2020.08.005 PMC7428749 38620595

[56] Niles J, Karnes H, Dlott J, Kaufman H. Association of ABO/Rh with SARS-CoV-2 positivity: the role of race and ethnicity in a female cohort. Am. J. Hematol. 96(1), E23–e26 (2021). 33064308 10.1002/ajh.26019 PMC7675235

[57] Latz CA, De Carlo C, Boitano L Blood type and outcomes in patients with COVID-19. Ann. Hematol. 99(9), 2113–2118 (2020). 32656591 10.1007/s00277-020-04169-1 PMC7354354

[58] Levi J, Telles P, Scrivani H, Campana G. Lack of association between ABO blood groups and susceptibility to SARS-CoV-2 infection. Vox Sang. 116(2), 251–252 (2021). 33103769 10.1111/vox.13015

[59] Boudin L, Janvier F, Bylicki O, Dutasta F. ABO blood groups are not associated with risk of acquiring the SARS-CoV-2 infection in young adults. Haematologica 105(12), 2841–2843 (2020). 33256383 10.3324/haematol.2020.265066 PMC7716357

[60] Dzik S, Eliason K, Morris E, Kaufman R, North C. COVID-19 and ABO blood groups. Transfusion 60(8), 1883–1884 (2020). 32562280 10.1111/trf.15946 PMC7323215

[61] Pendu J, Breiman A, Rocher J, Dion M, Nathalie R. ABO blood types and COVID-19: spurious, anecdotal, or truly important relationships? A reasoned review of available data. Viruses 13(2), 160 (2021). 33499228 10.3390/v13020160 PMC7911989

[62] Silva-Filho JC, Melo CGF, Oliveira JL. The influence of ABO blood groups on COVID-19 susceptibility and severity: a molecular hypothesis based on carbohydrate-carbohydrate interactions. Med. Hypotheses 144, 110155 (2020). 33254482 10.1016/j.mehy.2020.110155 PMC7395945

